# 1-[(2-Chloro­phen­yl)di­phenyl­meth­yl]-1*H*-pyrazole

**DOI:** 10.1107/S2414314624011520

**Published:** 2024-12-03

**Authors:** Pierre Koch, Dieter Schollmeyer

**Affiliations:** a Department of Pharmaceutical/Medicinal Chemistry II, Institute for Pharmacy, Universität Regensburg, Universitätsstr. 31, 93053 Regensburg, Germany; b University of Mainz, Department of Chemistry, Duesbergweg 10-14, 55099 Mainz, Germany; University of Aberdeen, United Kingdom

**Keywords:** crystal structure, TRAM-34

## Abstract

The title compound C_22_H_17_ClN_2_, also named as TRAM-34, crystallizes in the monoclinic space group *P*2_1_/n. The dihedral angles between the pyrazole ring and the three six-membered rings are 62.28 (9), 69.48 (9) and 71.30 (9)°.

## Graphical abstract

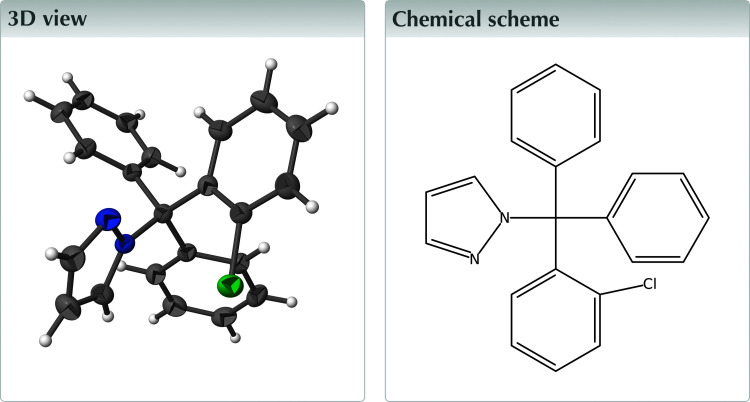

## Structure description

**Figure 1:**
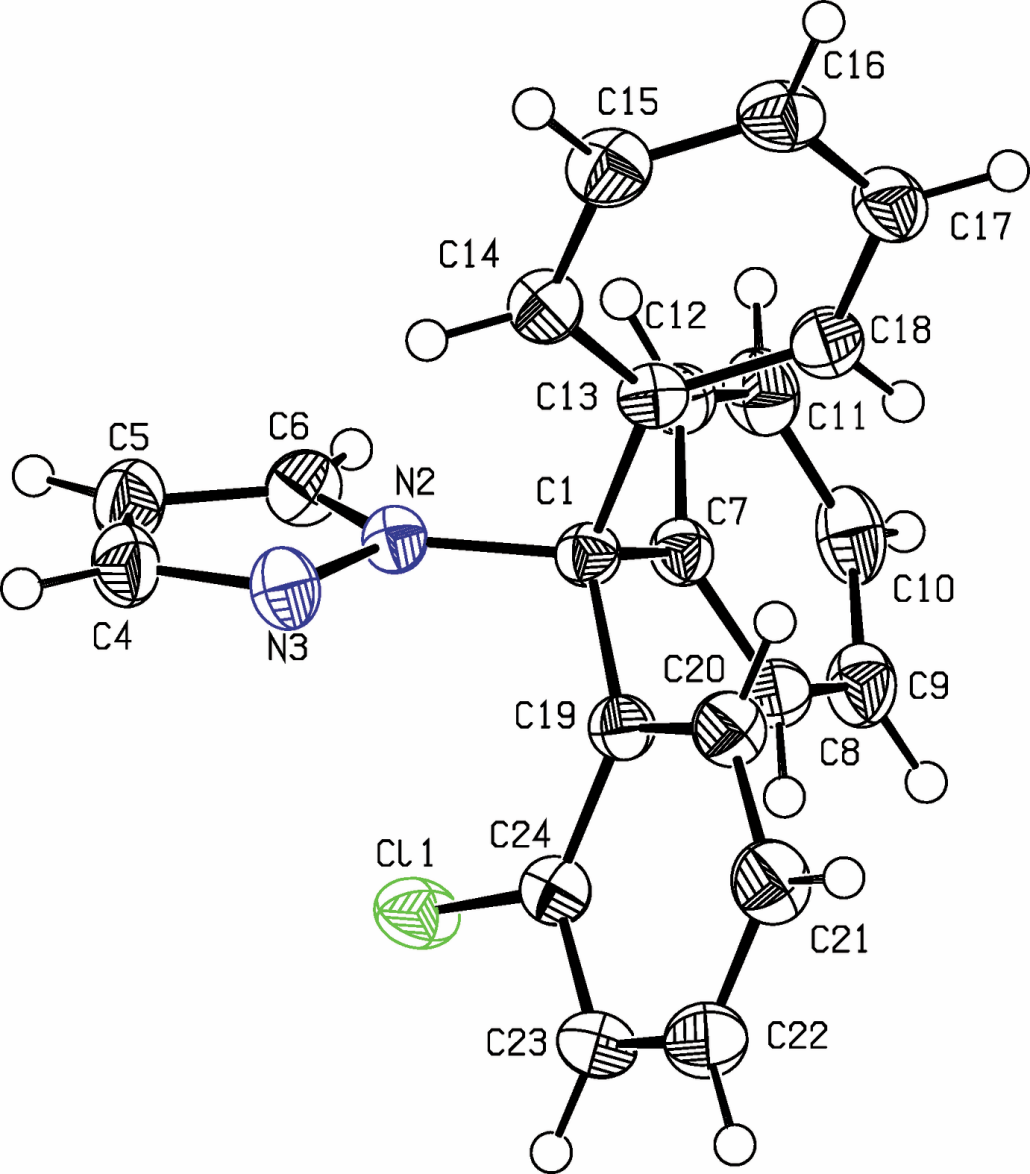
The mol­ecular structure of **I**. Displacement ellipsoids are drawn at the 50% probability level.

The title compound, C_22_H_17_ClN_2_ (**I**) Fig. 1 ▸, also named as tri­aryl­methane-34 (TRAM-34), is a structural isomer of the anti-fungal drug clotrimazole or 1-[(2-chloro­phen­yl)di­phenyl­meth­yl]-1*H*-imidazole. TRAM-34 is a selective and potent inhibitor of the inter­mediate-conductance, calcium-activated K^+^ channels *K*_Ca_3.1 (*K*_D_ = 20–25 n*M*) (Wulff *et al.*, 2000 ▸, 2001 ▸). TRAM-34 was synthesized and investigated in two studies to analyze the *in vivo* effect of combined irradiation and *K*_Ca_-targeting with TRAM-34 in a glioma mouse model (Stransky *et al.*, 2023 ▸; Ganser *et al.*, 2024 ▸).

**Figure 2:**
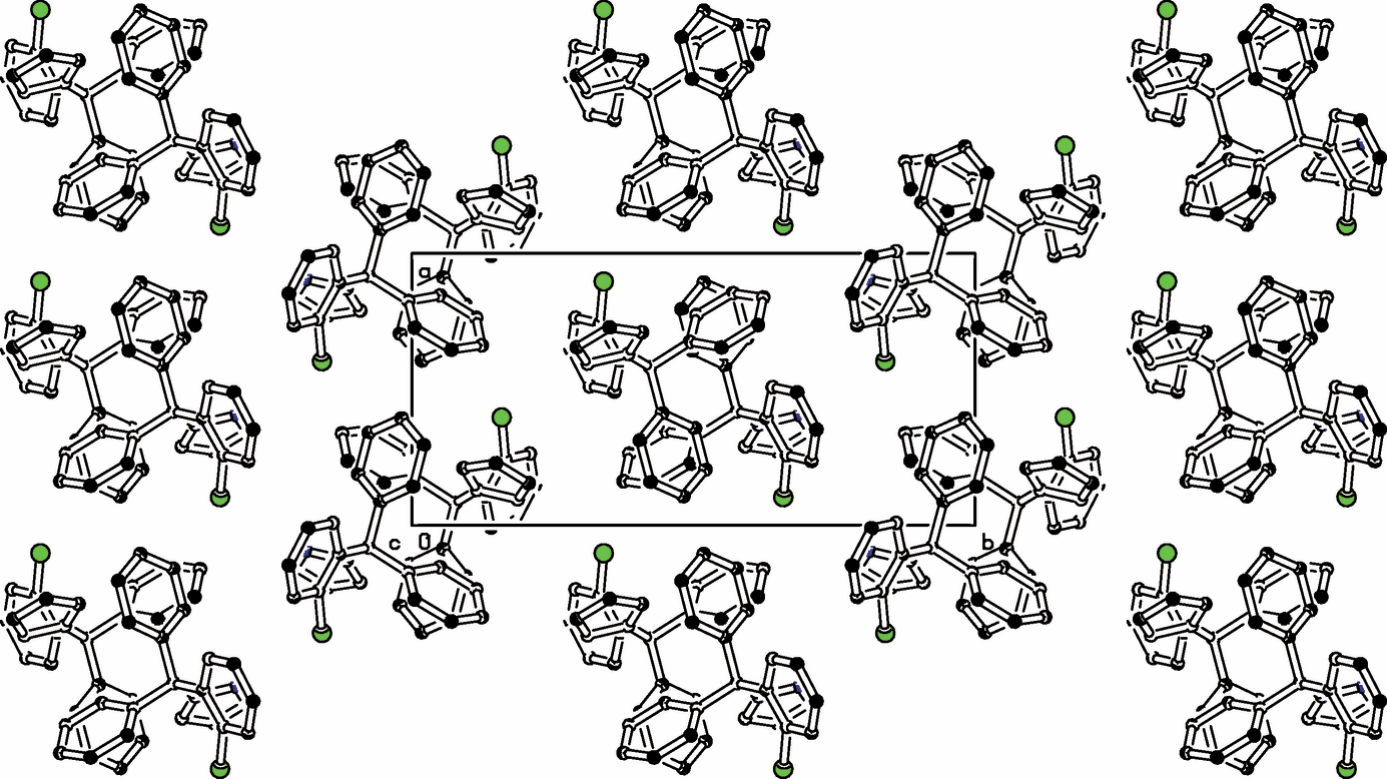
Part of the packing diagram viewed along the *c*-axis direction. Hydrogen atoms removed for clarity.

The dihedral angles in **I** between the pyrazole ring and the three six-membered rings (C7–C12, C13–C18, and C19–C24) are 62.28 (9), 69.48 (9), and 71.30 (9)°, respectively. The 2-chloro­benzene ring (C19–C24) is almost perpendicular to the C13–C18 ring [dihedral angle = 81.27 (7)°]. The dihedral angles between the C7–C12 ring and the C13–C18 and C19–C24 rings are 71.44 (8) and 69.05 (8)°, respectively. For the crystal structure of clotrimazole, see Song *et al.* (1998 ▸). In the extended structure of (**I**), some weak C—H⋯π inter­actions (Table 1 ▸) link the mol­ecules Fig. 2 ▸.

## Synthesis and crystallization

The title compound was prepared using the synthetic strategy reported by Wulff *et al.* (2000 ▸). To a suspension of 2-chloro­trityl chloride (12.5 g, 40 mmol) in aceto­nitrile (500 ml) was added pyrazole (8.17 g, 120 mmol). The reaction mixture was heated to reflux temperature for 3 h (during this time the reaction mixture became clear). After cooling to room temperature, the solvent was removed, and the residue was dissolved in ethyl acetate (200 ml). The organic phase was washed with water (3 × 150 ml). During this process, the title compound precipitated as a white solid, which was collected by filtration and dried (3.44 g, 25%). The filtrate was dried over sodium sulfate and solvent was evaporated. The obtained residue was recrystallized from hot ethanol solution to yield additional 7.11 g (52%) of the title compound as colorless crystals.

## Refinement

Crystal data, data collection and structure refinement details are summarized in Table 2 ▸.

## Supplementary Material

Crystal structure: contains datablock(s) I, global. DOI: 10.1107/S2414314624011520/hb4496sup1.cif

Structure factors: contains datablock(s) I. DOI: 10.1107/S2414314624011520/hb4496Isup2.hkl

Supporting information file. DOI: 10.1107/S2414314624011520/hb4496Isup3.cml

CCDC reference: 2405489

Additional supporting information:  crystallographic information; 3D view; checkCIF report

## Figures and Tables

**Table 1:** Hydrogen-bond geometry (Å, °) *Cg*2 and *Cg*3 are the centroids of the C7–C12 and C13–C18 rings, respectively.

*D*—H⋯*A*	*D*—H	H⋯*A*	*D*⋯*A*	*D*—H⋯*A*
C6—H6⋯*Cg*3^i^	0.94	2.93	3.5594 (16)	125
C16—H16⋯*Cg*2^ii^	0.94	2.88	3.6868 (18)	145
C20—H20⋯*Cg*3	0.94	2.89	3.6164 (17)	135

Symmetry codes: (i) 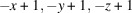; (ii) 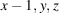.

**Table 2:** Experimental details

Crystal data	
Chemical formula	C_22_H_17_ClN_2_
*M*_r_	344.82
Crystal system, space group	Monoclinic, *P*2_1_/*n*
Temperature (K)	233
*a*, *b*, *c* (Å)	8.8768 (3), 18.3002 (7), 10.5053 (3)
β (°)	95.942 (3)
*V* (Å^3^)	1697.39 (10)
*Z*	4
Radiation type	Mo *K*α
μ (mm^−1^)	0.23
Crystal size (mm)	0.40 × 0.30 × 0.06

Data collection	
Diffractometer	Stoe IPDS 2T
No. of measured, independent and observed [*I* > 2σ(*I*)] reflections	22452, 4092, 2854
*R*_int_	0.070
(sin θ/λ)_max_ (Å^−1^)	0.661

Refinement	
*R*[*F*^2^ > 2σ(*F*^2^)], *wR*(*F*^2^), *S*	0.037, 0.089, 1.00
No. of reflections	4092
No. of parameters	226
H-atom treatment	H-atom parameters constrained
Δρ_max_, Δρ_min_ (e Å^−3^)	0.25, −0.26

Computer programs: *X-AREAWinXpose*, *Recipe* and *Integrate* (Stoe & Cie, 2019 ▸), *SHELXT2014* (Sheldrick, 2015*a* ▸), *SHELXL2018/3* (Sheldrick, 2015*b* ▸) and *PLATON* (Spek, 2020 ▸).

## full crystallographic data

### 1-[(2-Chlorophenyl)diphenylmethyl]-1H-pyrazole Crystal data

**Table:**

C_22_H_17_ClN_2_	*F*(000) = 720
*M**_r_* = 344.82	*D*_x_ = 1.349 Mg m^−^^3^
Monoclinic, *P*2_1_/*n*	Mo *K*α radiation, λ = 0.71073 Å
*a* = 8.8768 (3) Å	Cell parameters from 21301 reflections
*b* = 18.3002 (7) Å	θ = 2.2–28.5°
*c* = 10.5053 (3) Å	µ = 0.23 mm^−^^1^
β = 95.942 (3)°	*T* = 233 K
*V* = 1697.39 (10) Å^3^	Plate, colourless
*Z* = 4	0.40 × 0.30 × 0.06 mm

### 1-[(2-Chlorophenyl)diphenylmethyl]-1H-pyrazole Data collection

**Table:**

Stoe IPDS 2T diffractometer	2854 reflections with *I* > 2σ(*I*)
Radiation source: sealed Tube	*R*_int_ = 0.070
Graphite monochromator	θ_max_ = 28.0°, θ_min_ = 2.2°
Detector resolution: 6.67 pixels mm^-1^	*h* = −11→10
rotation method scans	*k* = −24→24
22452 measured reflections	*l* = −13→13
4092 independent reflections	

### 1-[(2-Chlorophenyl)diphenylmethyl]-1H-pyrazole Refinement

**Table:**

Refinement on *F*^2^	Primary atom site location: structure-invariant direct methods
Least-squares matrix: full	Hydrogen site location: inferred from neighbouring sites
*R*[*F*^2^ > 2σ(*F*^2^)] = 0.037	H-atom parameters constrained
*wR*(*F*^2^) = 0.089	*w* = 1/[σ^2^(*F*_o_^2^) + (0.0431*P*)^2^ + 0.0511*P*] where *P* = (*F*_o_^2^ + 2*F*_c_^2^)/3
*S* = 1.00	(Δ/σ)_max_ = 0.001
4092 reflections	Δρ_max_ = 0.25 e Å^−^^3^
226 parameters	Δρ_min_ = −0.26 e Å^−^^3^
0 restraints	

### 1-[(2-Chlorophenyl)diphenylmethyl]-1H-pyrazole Special details

**Table:**

Geometry. All esds (except the esd in the dihedral angle between two l.s. planes) are estimated using the full covariance matrix. The cell esds are taken into account individually in the estimation of esds in distances, angles and torsion angles; correlations between esds in cell parameters are only used when they are defined by crystal symmetry. An approximate (isotropic) treatment of cell esds is used for estimating esds involving l.s. planes.
Refinement. Hydrogen atoms attached to carbons were placed at calculated positions and were refined in the riding-model approximation with C—H = 0.95 Å, and with *U*_iso_(H) = 1.2 *U*_eq_(C).

### 1-[(2-Chlorophenyl)diphenylmethyl]-1H-pyrazole Fractional atomic coordinates and isotropic or equivalent isotropic displacement parameters (Å^2^)

**Table:**

	*x*	*y*	*z*	*U*_iso_*/*U*_eq_	
Cl1	0.89682 (4)	0.34060 (2)	0.81219 (4)	0.03752 (11)	
C1	0.58130 (17)	0.42292 (7)	0.75062 (13)	0.0250 (3)	
N2	0.62973 (14)	0.38408 (6)	0.63686 (11)	0.0254 (3)	
N3	0.59467 (16)	0.31210 (7)	0.62163 (12)	0.0325 (3)	
C4	0.6549 (2)	0.29284 (9)	0.51660 (15)	0.0360 (4)	
H4	0.648932	0.245476	0.481808	0.043*	
C5	0.7282 (2)	0.35090 (9)	0.46361 (16)	0.0399 (4)	
H5	0.778981	0.350618	0.389382	0.048*	
C6	0.71009 (19)	0.40817 (8)	0.54324 (14)	0.0326 (3)	
H6	0.746928	0.455872	0.534524	0.039*	
C7	0.67484 (16)	0.49371 (8)	0.77271 (13)	0.0257 (3)	
C8	0.77370 (17)	0.50538 (8)	0.88172 (14)	0.0297 (3)	
H8	0.786378	0.468877	0.944871	0.036*	
C9	0.85418 (19)	0.57016 (9)	0.89893 (16)	0.0370 (4)	
H9	0.920168	0.577367	0.973789	0.044*	
C10	0.8380 (2)	0.62395 (9)	0.80707 (17)	0.0391 (4)	
H10	0.895620	0.667054	0.817139	0.047*	
C11	0.7365 (2)	0.61397 (8)	0.70024 (16)	0.0384 (4)	
H11	0.723045	0.650950	0.638004	0.046*	
C12	0.65441 (18)	0.54996 (8)	0.68393 (15)	0.0316 (3)	
H12	0.583602	0.544385	0.611624	0.038*	
C13	0.41300 (17)	0.44410 (8)	0.72473 (13)	0.0259 (3)	
C14	0.31188 (18)	0.40494 (8)	0.64180 (15)	0.0314 (3)	
H14	0.346806	0.364934	0.597033	0.038*	
C15	0.15994 (19)	0.42380 (9)	0.62375 (16)	0.0380 (4)	
H15	0.093076	0.396539	0.566990	0.046*	
C16	0.10643 (19)	0.48194 (9)	0.68808 (15)	0.0367 (4)	
H16	0.003507	0.494899	0.675255	0.044*	
C17	0.20511 (19)	0.52101 (9)	0.77149 (15)	0.0353 (4)	
H17	0.169256	0.560687	0.816524	0.042*	
C18	0.35642 (18)	0.50244 (9)	0.78966 (15)	0.0323 (3)	
H18	0.422426	0.529778	0.847021	0.039*	
C19	0.60600 (17)	0.37049 (7)	0.86593 (13)	0.0262 (3)	
C20	0.49184 (19)	0.35937 (8)	0.94499 (15)	0.0324 (3)	
H20	0.397746	0.382274	0.924093	0.039*	
C21	0.5113 (2)	0.31593 (9)	1.05318 (16)	0.0387 (4)	
H21	0.431234	0.309744	1.104196	0.046*	
C22	0.6475 (2)	0.28186 (9)	1.08598 (16)	0.0391 (4)	
H22	0.661712	0.252793	1.160029	0.047*	
C23	0.7635 (2)	0.29062 (8)	1.00941 (15)	0.0348 (4)	
H23	0.856956	0.267262	1.030945	0.042*	
C24	0.74213 (18)	0.33379 (8)	0.90091 (14)	0.0292 (3)	

### 1-[(2-Chlorophenyl)diphenylmethyl]-1H-pyrazole Atomic displacement parameters (Å^2^)

**Table:**

	*U*^11^	*U*^22^	*U*^33^	*U*^12^	*U*^13^	*U*^23^
Cl1	0.0269 (2)	0.0464 (2)	0.0397 (2)	0.00709 (17)	0.00596 (15)	0.00740 (17)
C1	0.0244 (7)	0.0250 (7)	0.0252 (7)	−0.0008 (6)	0.0013 (6)	−0.0002 (5)
N2	0.0235 (6)	0.0254 (6)	0.0271 (6)	−0.0019 (5)	0.0025 (5)	−0.0019 (5)
N3	0.0331 (7)	0.0267 (6)	0.0383 (7)	−0.0044 (5)	0.0067 (6)	−0.0050 (5)
C4	0.0378 (9)	0.0329 (8)	0.0373 (9)	−0.0007 (7)	0.0040 (7)	−0.0091 (7)
C5	0.0488 (11)	0.0414 (9)	0.0308 (8)	0.0024 (8)	0.0113 (7)	−0.0018 (7)
C6	0.0341 (9)	0.0325 (8)	0.0317 (8)	−0.0032 (7)	0.0061 (6)	0.0031 (6)
C7	0.0228 (7)	0.0268 (7)	0.0276 (7)	0.0002 (6)	0.0032 (6)	−0.0028 (6)
C8	0.0266 (8)	0.0319 (7)	0.0301 (7)	0.0016 (6)	0.0001 (6)	−0.0033 (6)
C9	0.0294 (9)	0.0404 (9)	0.0406 (9)	−0.0016 (7)	0.0003 (7)	−0.0141 (7)
C10	0.0374 (10)	0.0307 (8)	0.0508 (10)	−0.0082 (7)	0.0129 (8)	−0.0135 (7)
C11	0.0469 (11)	0.0272 (8)	0.0425 (9)	−0.0027 (7)	0.0109 (8)	−0.0001 (7)
C12	0.0333 (9)	0.0289 (7)	0.0320 (8)	−0.0004 (6)	0.0009 (6)	−0.0001 (6)
C13	0.0235 (7)	0.0273 (7)	0.0268 (7)	−0.0017 (6)	0.0022 (6)	0.0025 (6)
C14	0.0276 (8)	0.0308 (7)	0.0353 (8)	−0.0036 (6)	0.0007 (6)	−0.0024 (6)
C15	0.0259 (8)	0.0453 (10)	0.0416 (9)	−0.0051 (7)	−0.0014 (7)	−0.0020 (7)
C16	0.0243 (8)	0.0464 (9)	0.0395 (9)	0.0022 (7)	0.0030 (7)	0.0067 (7)
C17	0.0304 (9)	0.0393 (8)	0.0370 (8)	0.0063 (7)	0.0077 (7)	0.0008 (7)
C18	0.0297 (8)	0.0344 (8)	0.0321 (8)	0.0003 (7)	0.0001 (6)	−0.0022 (6)
C19	0.0272 (8)	0.0237 (7)	0.0272 (7)	−0.0008 (6)	0.0013 (6)	−0.0003 (5)
C20	0.0322 (9)	0.0322 (8)	0.0337 (8)	0.0000 (7)	0.0075 (7)	0.0021 (6)
C21	0.0426 (10)	0.0378 (9)	0.0378 (9)	0.0005 (7)	0.0143 (8)	0.0049 (7)
C22	0.0504 (11)	0.0334 (8)	0.0339 (8)	0.0020 (8)	0.0063 (8)	0.0093 (7)
C23	0.0371 (9)	0.0306 (8)	0.0362 (8)	0.0059 (7)	0.0008 (7)	0.0053 (6)
C24	0.0282 (8)	0.0284 (7)	0.0307 (7)	−0.0004 (6)	0.0026 (6)	0.0002 (6)

### 1-[(2-Chlorophenyl)diphenylmethyl]-1H-pyrazole Geometric parameters (Å, º)

**Table:**

Cl1—C24	1.7420 (16)	C12—H12	0.9400
C1—N2	1.4915 (18)	C13—C14	1.384 (2)
C1—C13	1.540 (2)	C13—C18	1.389 (2)
C1—C19	1.5430 (19)	C14—C15	1.386 (2)
C1—C7	1.5433 (19)	C14—H14	0.9400
N2—C6	1.3475 (19)	C15—C16	1.371 (2)
N2—N3	1.3592 (17)	C15—H15	0.9400
N3—C4	1.323 (2)	C16—C17	1.374 (2)
C4—C5	1.392 (2)	C16—H16	0.9400
C4—H4	0.9400	C17—C18	1.379 (2)
C5—C6	1.361 (2)	C17—H17	0.9400
C5—H5	0.9400	C18—H18	0.9400
C6—H6	0.9400	C19—C20	1.390 (2)
C7—C8	1.385 (2)	C19—C24	1.398 (2)
C7—C12	1.388 (2)	C20—C21	1.383 (2)
C8—C9	1.386 (2)	C20—H20	0.9400
C8—H8	0.9400	C21—C22	1.373 (2)
C9—C10	1.376 (2)	C21—H21	0.9400
C9—H9	0.9400	C22—C23	1.380 (2)
C10—C11	1.377 (3)	C22—H22	0.9400
C10—H10	0.9400	C23—C24	1.384 (2)
C11—C12	1.381 (2)	C23—H23	0.9400
C11—H11	0.9400		
			
N2—C1—C13	109.46 (11)	C14—C13—C18	117.59 (14)
N2—C1—C19	107.66 (11)	C14—C13—C1	122.33 (13)
C13—C1—C19	110.58 (11)	C18—C13—C1	120.02 (13)
N2—C1—C7	108.98 (11)	C13—C14—C15	121.04 (15)
C13—C1—C7	108.19 (11)	C13—C14—H14	119.5
C19—C1—C7	111.92 (12)	C15—C14—H14	119.5
C6—N2—N3	111.30 (12)	C16—C15—C14	120.50 (15)
C6—N2—C1	130.33 (12)	C16—C15—H15	119.7
N3—N2—C1	118.34 (11)	C14—C15—H15	119.7
C4—N3—N2	104.41 (12)	C15—C16—C17	119.18 (16)
N3—C4—C5	112.11 (14)	C15—C16—H16	120.4
N3—C4—H4	123.9	C17—C16—H16	120.4
C5—C4—H4	123.9	C16—C17—C18	120.50 (15)
C6—C5—C4	104.60 (14)	C16—C17—H17	119.8
C6—C5—H5	127.7	C18—C17—H17	119.8
C4—C5—H5	127.7	C17—C18—C13	121.19 (15)
N2—C6—C5	107.58 (14)	C17—C18—H18	119.4
N2—C6—H6	126.2	C13—C18—H18	119.4
C5—C6—H6	126.2	C20—C19—C24	115.87 (13)
C8—C7—C12	117.94 (14)	C20—C19—C1	120.46 (13)
C8—C7—C1	122.59 (13)	C24—C19—C1	123.63 (13)
C12—C7—C1	119.37 (13)	C21—C20—C19	122.59 (15)
C7—C8—C9	120.89 (15)	C21—C20—H20	118.7
C7—C8—H8	119.6	C19—C20—H20	118.7
C9—C8—H8	119.6	C22—C21—C20	119.94 (15)
C10—C9—C8	120.37 (15)	C22—C21—H21	120.0
C10—C9—H9	119.8	C20—C21—H21	120.0
C8—C9—H9	119.8	C21—C22—C23	119.47 (15)
C9—C10—C11	119.26 (15)	C21—C22—H22	120.3
C9—C10—H10	120.4	C23—C22—H22	120.3
C11—C10—H10	120.4	C22—C23—C24	119.99 (15)
C10—C11—C12	120.41 (16)	C22—C23—H23	120.0
C10—C11—H11	119.8	C24—C23—H23	120.0
C12—C11—H11	119.8	C23—C24—C19	122.12 (14)
C11—C12—C7	121.00 (15)	C23—C24—Cl1	115.68 (12)
C11—C12—H12	119.5	C19—C24—Cl1	122.19 (11)
C7—C12—H12	119.5		
			
C13—C1—N2—C6	102.06 (17)	C7—C1—C13—C14	146.06 (14)
C19—C1—N2—C6	−137.68 (15)	N2—C1—C13—C18	−155.38 (13)
C7—C1—N2—C6	−16.1 (2)	C19—C1—C13—C18	86.16 (16)
C13—C1—N2—N3	−80.21 (15)	C7—C1—C13—C18	−36.74 (17)
C19—C1—N2—N3	40.04 (16)	C18—C13—C14—C15	0.5 (2)
C7—C1—N2—N3	161.64 (12)	C1—C13—C14—C15	177.75 (14)
C6—N2—N3—C4	−0.18 (17)	C13—C14—C15—C16	0.0 (2)
C1—N2—N3—C4	−178.31 (13)	C14—C15—C16—C17	−0.5 (2)
N2—N3—C4—C5	0.01 (19)	C15—C16—C17—C18	0.5 (2)
N3—C4—C5—C6	0.2 (2)	C16—C17—C18—C13	−0.1 (2)
N3—N2—C6—C5	0.28 (18)	C14—C13—C18—C17	−0.4 (2)
C1—N2—C6—C5	178.13 (14)	C1—C13—C18—C17	−177.77 (14)
C4—C5—C6—N2	−0.26 (19)	N2—C1—C19—C20	−131.44 (14)
N2—C1—C7—C8	−116.07 (15)	C13—C1—C19—C20	−11.89 (18)
C13—C1—C7—C8	124.98 (15)	C7—C1—C19—C20	108.82 (15)
C19—C1—C7—C8	2.90 (19)	N2—C1—C19—C24	50.99 (17)
N2—C1—C7—C12	67.44 (16)	C13—C1—C19—C24	170.54 (13)
C13—C1—C7—C12	−51.50 (17)	C7—C1—C19—C24	−68.75 (17)
C19—C1—C7—C12	−173.59 (13)	C24—C19—C20—C21	1.2 (2)
C12—C7—C8—C9	−2.5 (2)	C1—C19—C20—C21	−176.56 (14)
C1—C7—C8—C9	−179.07 (14)	C19—C20—C21—C22	0.0 (3)
C7—C8—C9—C10	−0.5 (2)	C20—C21—C22—C23	−0.8 (3)
C8—C9—C10—C11	2.6 (2)	C21—C22—C23—C24	0.3 (2)
C9—C10—C11—C12	−1.5 (3)	C22—C23—C24—C19	1.0 (2)
C10—C11—C12—C7	−1.6 (3)	C22—C23—C24—Cl1	179.98 (13)
C8—C7—C12—C11	3.6 (2)	C20—C19—C24—C23	−1.7 (2)
C1—C7—C12—C11	−179.73 (14)	C1—C19—C24—C23	175.98 (14)
N2—C1—C13—C14	27.42 (18)	C20—C19—C24—Cl1	179.37 (11)
C19—C1—C13—C14	−91.04 (16)	C1—C19—C24—Cl1	−3.0 (2)

### 1-[(2-Chlorophenyl)diphenylmethyl]-1H-pyrazole Hydrogen-bond geometry (Å, º)

*Cg*2 and *Cg*3 are the centroids of the C7–C12 and C13–C18 rings, respectively.

**Table:**

*D*—H···*A*	*D*—H	H···*A*	*D*···*A*	*D*—H···*A*
C6—H6···*Cg*3^i^	0.94	2.93	3.5594 (16)	125
C16—H16···*Cg*2^ii^	0.94	2.88	3.6868 (18)	145
C20—H20···*Cg*3	0.94	2.89	3.6164 (17)	135

Symmetry codes: (i) −*x*+1, −*y*+1, −*z*+1; (ii) *x*−1, *y*, *z*.
